# Peroxidase-like activity of biosynthesized silver nanoparticles for colorimetric detection of cysteine†

**DOI:** 10.1039/d3ra01587d

**Published:** 2023-05-31

**Authors:** Melisew Tadele Alula

**Affiliations:** a Department of Chemical and Forensic Sciences, Faculty of Science, Botswana International University of Science and Technology Plot 10071, Private Bag 16 Palapye Botswana alulam@biust.ac.bw +267-4900102 +267-76126741

## Abstract

Cysteine is one of the important amino acids that is involved in various physiological processes, food industries, pharmaceuticals, and personal care. It also serves as a biomarker for some diseases. The large use of cysteine necessitates rapid, cheap, and accurate determination of cysteine in a range of samples. Although many techniques have been employed for the detection of cysteine, they suffer from limitations that make them unsuitable for routine analysis. Here we report on a cheap colorimetric method using biosynthesized silver nanoparticles (AgNPs) as nanozymes. The AgNPs were characterized by UV/visible spectrophotometry, scanning electron microscopy (SEM), and surface-enhanced Raman spectroscopy (SERS). The AgNPs exhibit peroxidase-like activity using *o*-phenylenediamine (OPD) as a chromogenic reagent. The low *K*_m_ values observed for OPD and H_2_O_2_ (0.9133 and 61.56 mM respectively) show strong affinity of the substrates to AgNPs. The peroxidase-like activity of AgNPs, however, was inhibited on the addition of cysteine. The results show that the absorption intensity of the oxidized OPD decreased linearly with the concentration of cysteine in the range of 0.5–20 μM. The limit of detection (LOD) in this linear range was found to be as low as 90.4 nM. The recovery from urine sample (spiked with cysteine) analyses demonstrated the feasibility of the method in real sample application. From our findings, we anticipate that our method can be applied for the analysis of cysteine in various samples.

## Abstract 2

The peroxidase-like activity of the biosynthesized AgNPs was inhibited on addition of cysteine. This enables us to develop a colorimetric method for cysteine detection.

## Graphical abstract

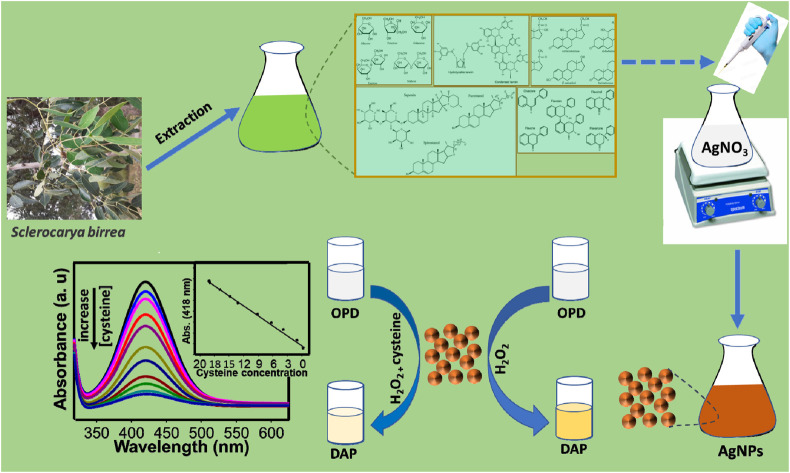

## 1. Introduction

Cysteine is one of the important amino acids and is known by its –SH group. Its functions in physiological processes are immense. These include protein folding and metabolism, posttranslational modification, and detoxification. Interestingly, its function as a biomarker related to many diseases makes cysteine vital in diagnosing many pathological ailments.^1^ Its utilization in industries of food, pharmaceuticals, and personal care is significant. The thiol group of cysteine is susceptible to oxidation so the resulting disulfide cysteine plays a significant role in structures of proteins. The thiol group also acts as an intracellular antioxidant and protects cells from damage.^2^ The large use of cysteine necessitates rapid, cheap, and accurate determination of cysteine in a range of samples.

Although a large number of techniques including electrochemistry,^3^ fluorescence spectroscopy,^4^ chemiluminescence,^5^ and chromatography^6^ have been employed for detection of cysteine, they suffer with limitations that make them unsuitable for routine analysis of cysteine. Colorimetric methods on the other hand reduce those limitations and have added advantages of low cost, rapid response, utilization of easy to operate instrument. Thus, they are suitable for routine analysis of different analytes of significant role, like cysteine. Colorimetric detection of cysteine based on the localized surface plasmon resonance (LSPR) properties of gold (AuNPs) and silver nanoparticles (AgNPs) have been reported intensively.^2,7–9^ The detection systems are based on aggregation or etching of AgNPs due to the target analytes. However, these necessitate careful surface functionalization or utilization of cross-linking agents.^2,10–12^ Therefore, the successful colorimetric detection of cysteine based on LSPR requires critical monitoring of the interactions of AgNPs and cysteine.^2^ This can reduce the quality of the detection system. Alternatively, colorimetric methods based on the catalytic activity of plasmonic nanoparticles were reported for detection of target analytes.^13,14^ The catalytic activity of the nanoparticles can be inhibited or promoted in the presence of the target analytes and the signals are tuned by concentrations of the target analytes. Herein we report an alternative colorimetric method based on the catalytic activity (enzyme-mimic) of AgNPs instead of its plasmonic property that avoids problems associated with the LSPR detection system of AgNPs.

Recently, colorimetric methods using nanomaterials with enzyme-mimic activities (nanozymes), as alternatives to the LSPR approach, have received intense attention in detection of various analytes.^15^ Nanozymes mimic the function of natural enzymes with the added advantage of low cost, high stability, and surface tunability.^15–17^ Nanozymes, like the natural enzymes, catalyze the conversion of substrates to oxidized colored products. By keeping the concentration of the substrate constant, it is possible to develop a colorimetric method where the intensity of the colored products can be analyzed by the naked eyes or by absorption spectrophotometer.^18^ Measurement of the intensity of the colored oxidized substrate can be correlated with the concentration of the target analytes and hence the colorimetric measurement is done. Since 2007 after the discovery^19^ of the intrinsic peroxidase-like activity of Fe_3_O_4_, colorimetric methods using various nanozymes have been employed intensively.^15^ Various nanomaterials including metal oxides,^20,21^ metal–organic framework,^22^ carbon-based materials,^23^ hybrids materials^13,24^ and metals^25–29^ have shown enzyme-mimic activities and employed for determination of analytes of different interest.

Among these, the enzyme catalytic activities of noble metal nanoparticles have received great attention in biochemical analysis and their activities are usually affected by sizes.^30^ Although AgNPs are cheaper as compared to other noble metal nanoparticles, synthesis of AgNPs with enzyme-mimic activities for analytical purposes is still not progressing well.^28,30^ Easy oxidation and controlling of the sizes of AgNPs contribute at large.^30^ Synthesis of stable AgNPs of high catalytic activities as nanozymes based detection is highly demanding.

Small organic ligands of molecules from natural resources are usually used as a reducing and capping agent. The reductive biomolecules and metabolites in plant extracts are involved in reduction of metal ions. Easy availability of the plants makes synthesis of silver nanoparticles using plant extracts sustainable and practical.^31^ Here, well dispersed stable AgNPs were prepared at room temperature following a green method using *Sclerocarya birrea* (morula) leaves extract. The produced AgNPs exhibits peroxidase-like activity using OPD as peroxidase substrate. The peroxidase-like activity of AgNPs using OPD as a chromogenic substrate, however, obstructed in the presence of cysteine. This enables us to develop a colorimetric method. The detection system is based on the inhibition of the peroxidase-like activity of AgNPs in the presence of cysteine.

## 2. Experimental

### 2.1. Chemicals

Chemicals of analytical grade reagents were used. Cysteine, glutathione, ascorbic acid, arginine, glycine, silver nitrate, hydrogen peroxide, lysine, phenylalanine, creatinine, uric acid, disodium hydrogen phosphate, sodium acetate, *o*-phenylenediamine (OPD), 4-aminothiophenol (4-ATP), and acetic acid were purchased from Sigma-Aldrich. Citric acid was purchased from Minima. Thiocyanate, and urea were obtained from Rochelle Chemicals.

### 2.2. Instrumentation

The UV/visible absorption spectra were gathered from UV/visible spectrophotometer (Thermo-Fisher). Quartz cuvette of 1 cm path length were used as sample cell. The SEM image was obtained from a focused ion beam scanning electron microscope (FIB-SEM) with STEM detector at primary 30 kV (Helios 5 UX, from Thermo Fisher Scientific). The XRD patterns of the synthesized AgNPs was collected from a D2 phaser XRD-300 W powder diffractometer (Bruker, AXS GmbH, Karlsruhe, Germany). SERS spectra were collected using the Horiba Labram Raman spectrometer of 532 nm laser line (JDS Uniphase Corporation, Milpitas, CA) and a CCD detector (Jobin-Yvon, Inc.). A Bruker FTIR spectrometer (specs (vertex 70v vacuum FTIR)) was used to collect the IR spectra.

### 2.3. Collection of morula leaves

*Sclerocarya birrea* (morula) leaves were collected from the surrounding area, Palapye, Botswana and cleaned thoroughly with distilled water. After removal of the debris and soil, the leaves dried in open air. The dried leaves were grounded to fine powder and kept in a glass bottle. The leaves extract was prepared as follows: 2 g of the leaves powder was transferred to a 40 mL deionized water and allowed to boil for 5 min. After it was cooled naturally, it was filtered using a Whatman paper and the extract was used to synthesize AgNPs.

### 2.4. Preparation of AgNPs

**Scheme 1:**
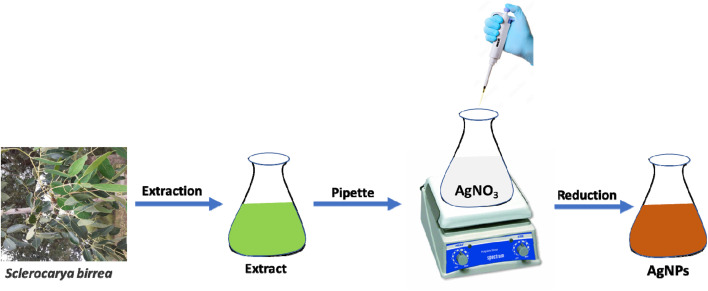
Synthesis of AgNPs.

AgNPs was prepared based on our previously reported method.^25^ A 200 mL of 5 mM AgNO_3_ solution in a conical flask was stirred vigorously at room temperature. To this solution, a 200 μL of the extract was added slowly and the color of the solution changed to red-brown quickly. The stirring continued at room temperature for 3 h and the synthesized AgNPs colloids were kept in a fridge. Scheme 1 shows the synthesis of AgNPs.

### 2.5. Peroxidase-like activity of AgNPs

The peroxidase-like activity of the synthesized AgNPs was evaluated using OPD as a chromogenic peroxidase substrate. In a typical reaction system, 25 μL of AgNPs, 800 μL of citrate–phosphate buffer (pH 6.2), 800 μL of 5 mM OPD, and 10 μL 4.9 M H_2_O_2_ were incubated for 20 min by shaking at 130 rpm on a shaker. The colorless solution changed to yellow that shows the peroxidase-like activity of the AgNPs. The steady-state kinetics of the peroxidase-like reactions were further studied to evaluate the peroxidase-like activity of the AgNPs. The kinetics of our synthesized AgNPs were studied by changing the concentrations of either OPD or H_2_O_2_ at a time but keeping the other constant.

### 2.6. Detection of cysteine

The peroxidase-like activity of the AgNPs was used to detect cysteine. The cysteine detection system was conducted based on the obstruction of the peroxidase-like activity of AgNPs in the presence of cysteine. For detection of cysteine, 25 μL of AgNPs, 800 μL of citrate–phosphate buffer (pH 6.2), 800 μL of 5 mM OPD, 10 μL 4.9 M H_2_O_2_, and 200 μL of cysteine of specified concentration were incubated on the shaker at 130 rpm. The UV/visible absorption spectroscopy measurements were conducted after 20 min of incubation.

## 3. Results and discussion

### 3.1. Synthesis and characterization of silver nanoparticles

**Fig. 1:**
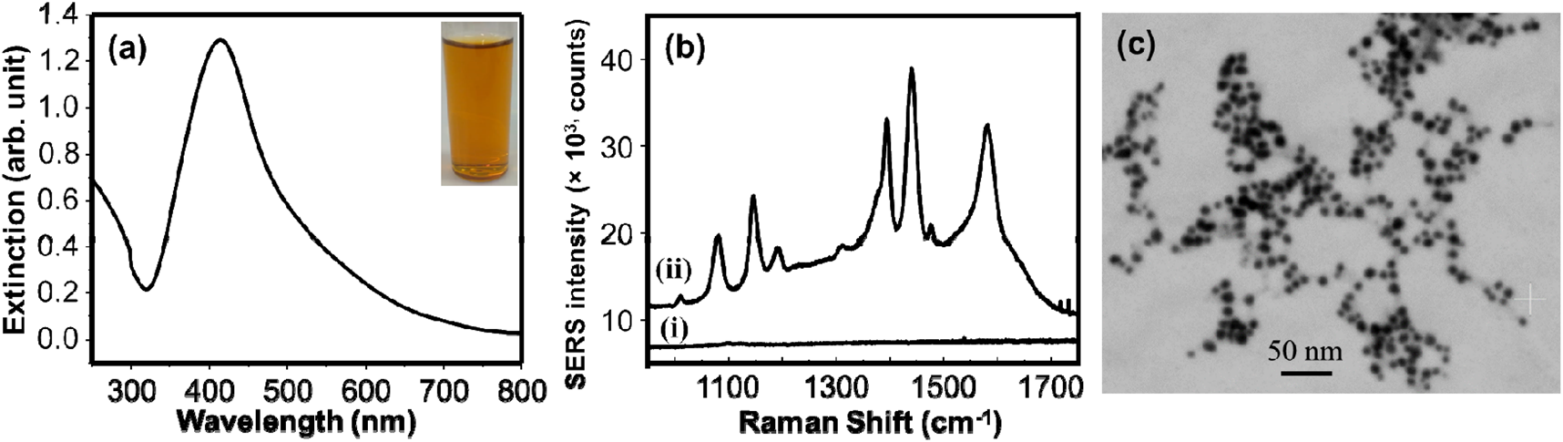
(a) Extinction spectrum of AgNPs colloids (the inset is the picture of the AgNPs colloids). (b) Raman spectra of 4-ATP using untreated filter paper (i) and filter paper impregnated in AgNPs colloids (ii) as SERS substrates. (c) SEM image of AgNPs.

A simple green wet chemical method was established to synthesize AgNPs using extracts of the leaves of *Sclerocarya birrea*. The red-brown colored colloids (Fig. 1a inset picture) were formed immediately after addition of the aqueous extract to the AgNO_3_ solution at room temperature showing the reducing capacity of the extract. Qualitative tests were investigated to evaluate the molecules responsible for the reduction of silver ions. The results (Table S1†) demonstrated the presence of steroids, terpenoids, saponins, sugars, flavonoids, and tannins. These are the commonly available chemicals in several plant leaves extract.^32,33^ These bioactive components possess strong reducing property and exhibit a high tendency to chemisorb on the surfaces of nanoparticles.^33,34^ The functional groups of the leaves extract that may involve in the synthesis of AgNPs were also investigated by FTIR (ATR mode). The FTIR measurements for both the extract and the AgNPs were conducted, and the spectra are given in Fig. S1a.† The similarity of the IR spectra of AgNPs and the extract may show the adsorption of organic molecules on the surface of AgNPs and functioned to stabilize the AgNPs. The broad band around 3334 cm^−1^ and the strong band at 1594 cm^−1^ may be attributed to –OH stretching vibrations^35^ and the hydrogen bonded NH groups of amides or amines.^36^ The bands around 1431 cm^−1^, 1367 cm^−1^, and 1047 cm^−1^ may be assigned to the aromatic ring C=C stretching, the C–N stretching and C–O stretching vibrations respectively.^37,38^ The presence of lone pairs of electrons that may be available on nitrogen or oxygen atoms of the amine/amide and the –OH functional groups can be donated to Ag^+^ ions with subsequent reduction of silver ions to Ag^0^.^35^ UV/visible absorption spectroscopy measurement was conducted to examine the red-brown colloids. The resulting surface plasmon resonance band (Fig. 1a) with *λ*_max_ at 414 nm shows the formation of colloidal AgNPs.

Since the discovery of anomalous enhancement of Raman signals for pyridine molecules adsorbed on the roughened silver electrode,^39^ enhancement of Raman signals (SERS) using plasmonic nanoparticles have received great attention. They have been tremendously used for sensitive detection of different Raman active molecules from various samples.^40,41^ Herein, we used SERS indirectly for examining the formation of AgNPs. AgNPs were adsorbed on the surface of Whatman filter paper simply by impregnating it in the synthesized colloidal AgNPs for 3 h. The filter paper was rinsed to remove loosely adsorbed AgNPs and dried before it had been used as a SERS substrate. Into a 1 mM solution of 4-ATP the dried impregnated filter paper and unmodified filter paper were soaked for 1 h before SERS measurements were done. The extremely enhanced Raman signal of 4-ATP from the impregnated filter paper was attributed to the AgNPs that are deposited on the filter paper that innate the SERS effect. The relatively medium peaks at 1076 cm^−1^ can be assigned to the C–S stretching vibrations. The 1143 cm^−1^ can be due to the C–N stretching and C–H bending vibrations. The band at 1186 cm^−1^ may be attributed to the N–N stretching vibrations. The N=N stretching vibrations may be responsible for the strong bands at 1381 cm^−1^ and 1438 cm^−1^. The prominent band at 1580 cm^−1^ could be assigned to the benzene ring C=C stretching vibrations.^42,43^ No noticeable Raman peaks were observed for the non-impregnated filter paper. This confirms that enhanced Raman signal is only due to the AgNPs. The size distribution and shape of the AgNPs were evaluated using SEM (Fig. 1c). The image shows synthesis of uniformly distributed AgNPs. This may be attributed to the various functional groups from the leaves extract that were functioning as capping agents.

### 3.2. Peroxidase-like activity of AgNPs and its application for detection of cysteine

**Fig. 2:**
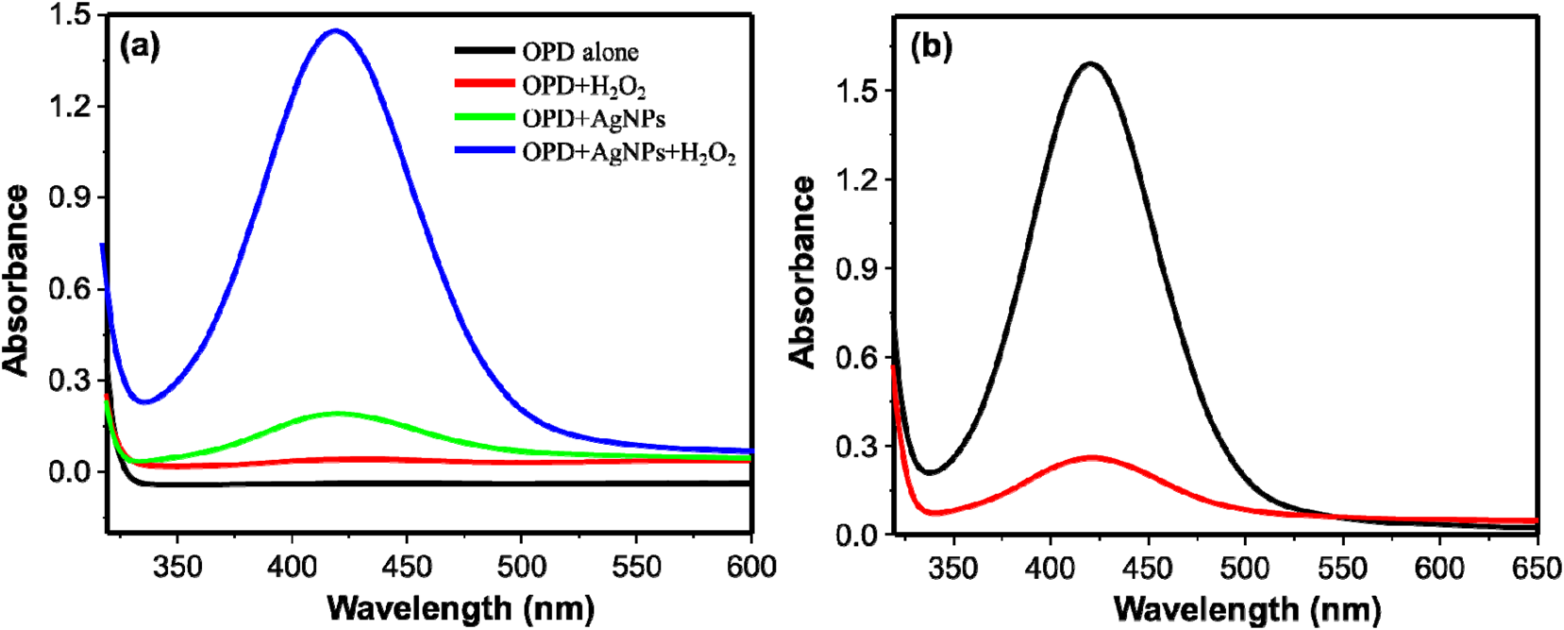
(a) UV/visible absorption spectra of OPD alone; OPD + H_2_O_2_; OPD + AgNPs; and OPD + AgNPs + H_2_O_2_. (b) UV/visible absorption spectra of DAP with (red curve) and without cysteine (black curve).

The peroxidase-like activity of the synthesized AgNPs was investigated using OPD as peroxidase substrate. OPD is one of the substrates used for investigating the peroxidase-like activity of nanozymes. Nanoparticles with the peroxidase-like activity oxidize the colorless OPD to the colored 2,3-diaminophenazine (DAP).^44^ Thus, four reaction systems using citrate–phosphate buffer (pH 6.2) as the reaction medium were studied: (i) OPD alone, (ii) OPD and H_2_O_2_, (iii) OPD and AgNPs (iv) OPD, H_2_O_2_, and AgNPs. Reaction systems (i) and (ii) didn't show any observable color change (Fig. 2a, black and red curves respectively). The color change of the reaction system from colorless to faint yellow and the corresponding absorption spectrum (Fig. 2a, green curve) for reaction system (iii) shows oxidation of OPD to DAP. This could be due to the oxidase-like activity of AgNPs.^25^ Remarkably, rapid formation of yellow colored solution from reaction system (iv) demonstrated the oxidation of OPD to DAP *via* peroxidase-like activity of AgNPs. The corresponding absorption spectra obtained from these reaction systems are shown on Fig. 2a. The formation of DAP through the peroxidase-like activity of AgNPs, however, was obstructed on addition of cysteine to the reaction system. The intensity of obstruction was reliant on the concentration of cysteine. This gave us an opportunity to develop a method for detection of cysteine. Fig. 2b shows the spectra obtained from reaction systems comprising of OPD, H_2_O_2_, and AgNPs in the absence (control) (black curve) and presence of cysteine (red curve).

### 3.3. Factors affecting the peroxidase-mimic performance of AgNPs

**Fig. 3:**
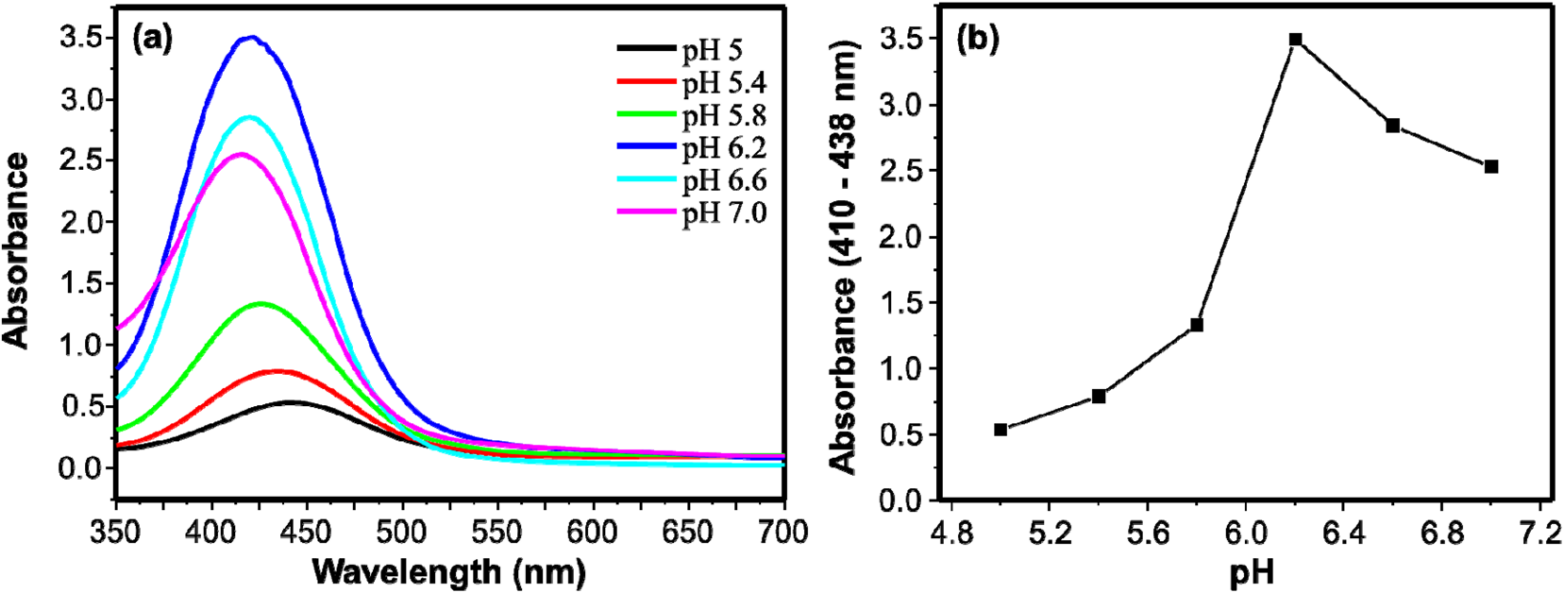
(a) UV/visible absorption spectra of DAP using citrate–phosphate buffers of different pH. (b) Relation between the absorbance and pH for the peroxidase-like activity of AgNPs.

The performances of peroxidase-like activities are affected by different reaction conditions. In this study, to achieve optimum performance of the peroxidase-mimic activity of the AgNPs, pH, catalyst loading, and reaction time were studied. Citrate–phosphate buffers in a pH range of 5.0 to 7.0 were studied. The catalytic activity increased with increasing pH from 5.0 to 6.2 and optimum performance was found at a pH of 6.2 (Fig. 3). The activity in turn started to decrease as the pH increased to 7.0. As shown in Fig. 3a, when the pH was increasing from 5.0 to 7.0, the maximum absorption shifted to shorter wavelength from 440 nm for pH 5.0 to 412 nm for pH 7.0. The variation in *λ*_max_ can be associated with the two p*K*_a_ values of the protonated forms of DAP.^45^

**Fig. 4:**
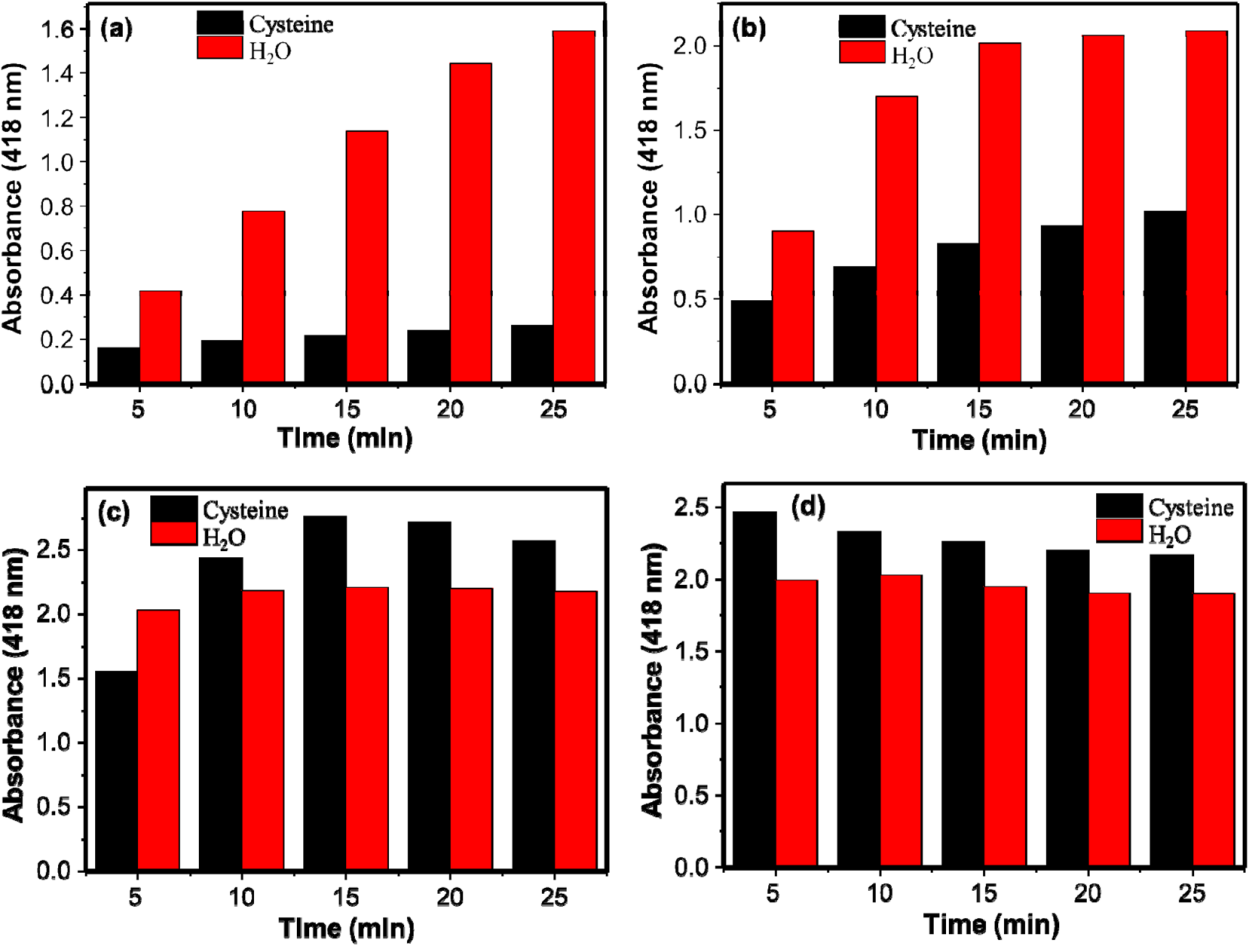
Relationship between absorbance and amount of catalyst (a) 10 μL, (b) 25 μL, (c) 50 μL, and (d) 75 μL on the peroxidase-like activity of AgNPs with and without cysteine.

The effect of the catalyst loading was studied both for the reaction systems containing cysteine and water (control) separately. The prepared AgNPs colloidal of volumes 10, 25, 50, and 75 μL were investigated and the absorption intensities with the catalyst load at 5, 10, 15, 20, and 25 min are given in Fig. 4. The corresponding spectra are given in Fig. S2.† For 5 min reaction time, the peroxidase-like activity of AgNPs increased with the catalyst load. For the 10 μL of the AgNPs, the formation of the oxidized product increased with time. For the 25 μL of AgNPs, on the other hand, the reaction did not proceed after 15 min of reaction time. The reaction progress was almost negligible after 5 min of reaction for 50 and 75 μL of AgNPs. As more active sites are available for the higher catalytic load, the reactions are completely within a short period of time. The performances of catalyst loading in the presence of cysteine were also studied to select the catalyst loading with high sensitivity in detection of cysteine; the larger difference in the absorbances of DAP in the presence and absence (water as a control) of cysteine implies the more sensitive the system would be. The difference in the absorbances for the control and cysteine for 10 μL of AgNPs increased with time. Whereas for 25 μL, initially it increased and started to decrease after 15 min of incubation showing the decrement in the inhibition capacity of cysteine. Interestingly for 50 and 75 μL of AgNPs, cysteine showed no inhibition effect at 10 min and 5 min reaction time respectively. On the contrary, in the presence of cysteine, the oxidation of OPD increased as shown on Fig. 4c and d. This may be associated with the mild aggregation of AgNPs by cysteine. Thiol-containing molecules could induce aggregation of metallic nanostructures. The aggregation of the metallic nanostructures changes the size of the nanoparticles and enhanced the peroxidase-like activity.^14^ Therefore, catalyst loading affects not only the peroxidase-like activity of AgNPs but also the sensitivity of the system in detection of cysteine. In this study 10 μL of AgNPs was used as optimum catalyst loading.

### 3.4. Kinetics study

**Fig. 5:**
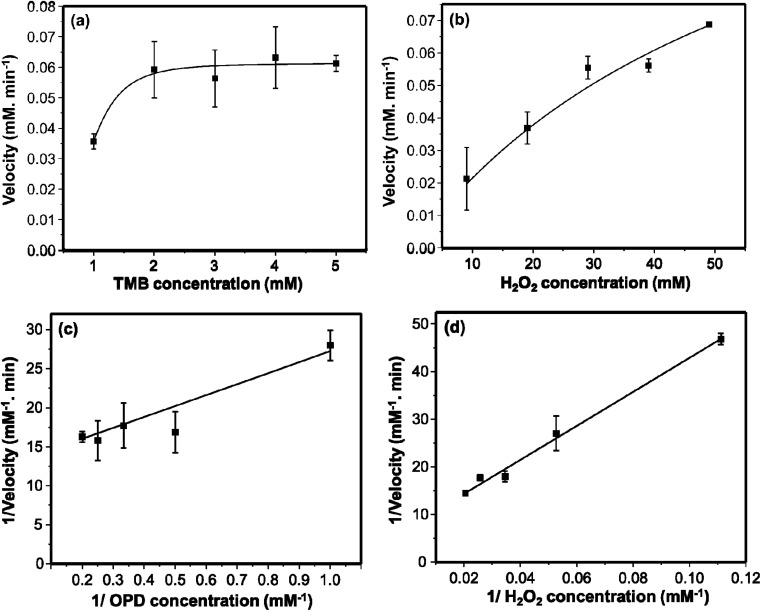
The Michael–Menton plots of TMB (a) and H_2_O_2_ (b). The Lineweaver–Burk double reciprocal plots of TMB (c) and H_2_O_2_ (d). 10 μL of AgNPs were used.

The kinetic assay was investigated further to evaluate the peroxidase-like activity of the AgNPs. The fact that the rate of catalysis of many enzymes depends on the concentrations of the substrates, the kinetics of our synthesized AgNPs were studied by changing the concentrations of either OPD or H_2_O_2_ at a time but keeping one constant. The resulting rate of catalysis was explained using Michaelis–Menten model as it is usually employed to explain the kinetics of enzymes. Fig. 5a and b show the Michaelis–Menten curves obtained for OPD and H_2_O_2_ respectively. Fig. 5c and d show the corresponding double-reciprocal Lineweaver–Burk plots. The *K*_m_ value is usually employed to evaluate the binding affinity of substrates towards enzymes; low *K*_m_ value shows strong binding affinity of the substrate towards the catalyst.^25^ Therefore, the relatively lower *K*_m_ values, 0.9133 mM for OPD and 61.56 mM for H_2_O_2_, show strong affinity of both substrates towards AgNPs. The *K*_m_ value for OPD is much lower than the previously reported, AgNPs@Fe_3_O_4_ (2.91 mM)^13^ and NiFe_2_O_4_ (8.4 mM)^46^ nanozymes.

### 3.5. Determination of cysteine

**Fig. 6:**
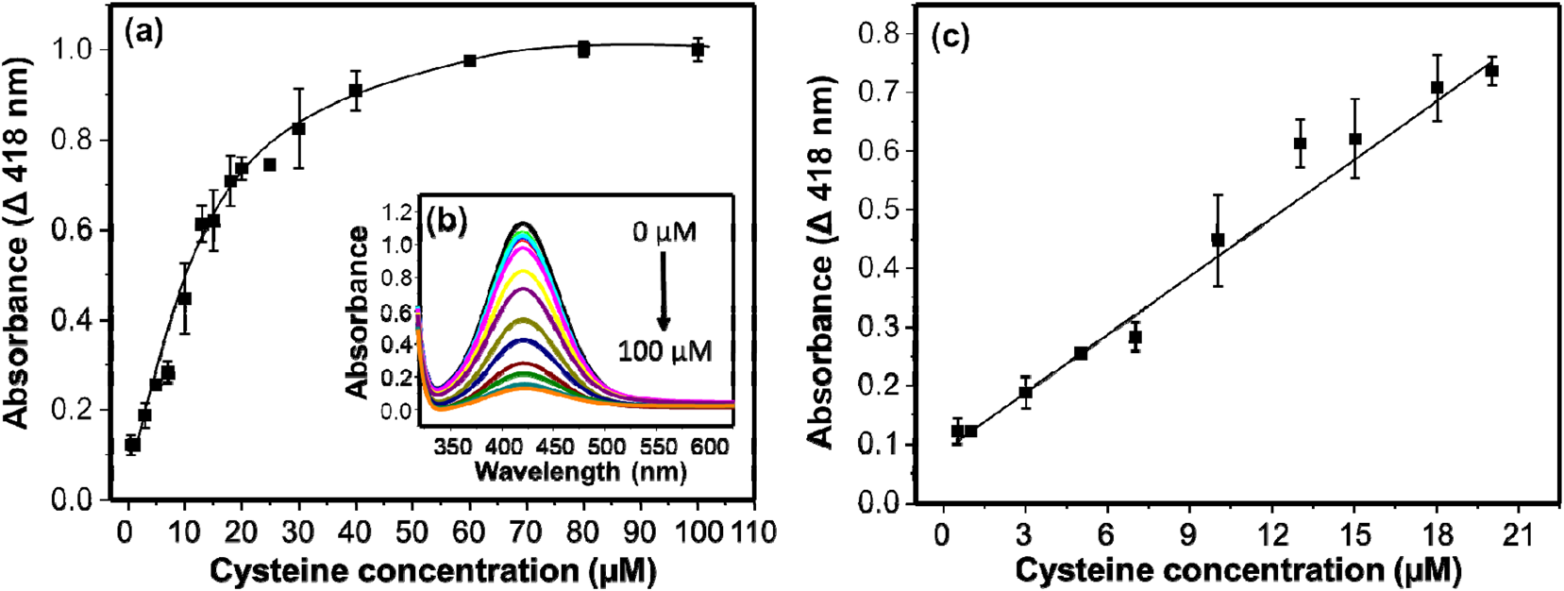
(a) Concentration profile curve for the determination of cysteine. (b) The UV/visible absorption spectra of DAP at different concentrations of cysteine. (c) Concentration–absorbance plot in linear range.

The detection of cysteine in this system is established on the inhibition of the AgNPs-promoted oxidation of OPD. The AgNPs-promoted oxidation of OPD to DAP has been inhibited in the presence of cysteine. The extent of inhibition is concentration dependent. In a specific reaction mixture, 750 μL of citrate–phosphate buffer (pH 6.2), 500 μL of cysteine in a range of concentrations (0–100 μM), 750 μL of 5 mM OPD, and 10 μL of 9.8 M H_2_O_2_ were incubated for 20 min on a shaker (130 rpm). The resulting UV/visible absorption spectra are given in Fig. 6b. The intensities decreased with increasing the concentration of cysteine. The inhibition of the catalytic activity may be associated with the thiophilic property of gold and silver nanomaterials towards thiol-containing molecules.^47–49^ The intensities decreased linearly in the concentration range of 0.5–20 μM. Then after, the intensities decreased gradually, and no significant change in absorption intensities were observed in a concentration range of 40–100 μM (Fig. 6a). The relation between Δ*A* (418 nm) and concentration of cysteine linearly fit with an equation *y* = 0.0332*x* + 0.0898 (*R*^2^ = 0.992). The limit of detection (LOD) was calculated using 3 times the standard deviation (3*σ*) and slope (*S*) of the linear equation and found as small as 0.0904 μM (90.4 nM). On Table 1, the LOD and linearity of colorimetric methods used for the determination of cysteine are given. Comparatively, our method exhibited low LOD and wide linearity.

**Table 1:** Comparison of previously reported nanozymes used in determination of cysteine with this study

No.	Nanomaterial	Limit of detection (μmol L^−1^)	Linear range (μmol L^−1^)	Reference
1	AgNPs@Fe_3_O_4_	0.087	0–20	13
2	DNA–Ag/Pt	0.002	0.005–0.5	47
3	Fe^3+^ ions	0.97	0–50	50
4	Fe, N-GQDs	0.14	0.5–50	51
5	Cu@Au(Ag)/Pt	4.0	0–400; 400–3000	52
6	AgNPs	0.0904	0.5–20	This work

**Fig. 7:**
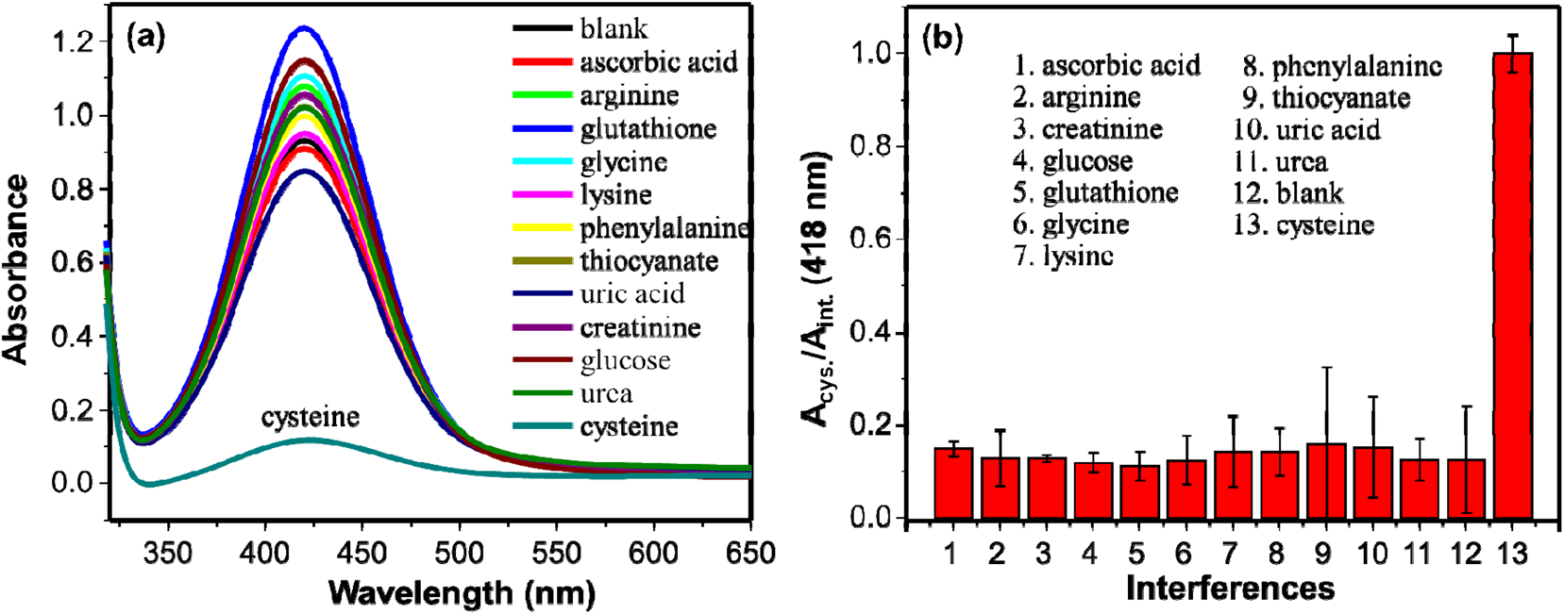
(a) UV/visible absorption spectra of DAP in the presence of various molecules. (b) The ratio of intensities (*A*_cysteine_/*A*_interference_) of DAP spectra (from (a)) using different molecules. The tested molecules are ascorbic acid, arginine, creatinine, glucose, glutathione, glycine, lysine, phenylalanine, thiocyanate, uric acid, urea, and cysteine.

Several other molecules were employed in place of cysteine for the reaction system to study the selectivity of the method. Molecules such as ascorbic acid, arginine, creatinine, glucose, glutathione, glycine, lysine, phenylalanine, thiocyanate, uric acid, and urea were tested. The concentrations of the tested molecules were 40 μM whereas that of cysteine was 20 μM which is two times less than the interferent molecules. The peroxidase-like activity of AgNPs remained practically uninhibited in the presence of the interferent molecules as shown on Fig. 7. The strong interaction of AgNPs and cysteine may inactivate the peroxidase-like activity of AgNPs. The extinction spectra of AgNPs with and without cysteine were measured to investigate the interaction of cysteine and AgNPs. As shown on Fig. S1b,† in the presence of cysteine, widening of the bandwidth and the *λ*_max_ shifts to longer wavelength. This shows interaction of cysteine with AgNPs.^53^ This interaction results in inhibitory effect on the catalytic activity of AgNPs. Similar observation has been reported by Liyanage *et al.*, (2021)^54^ such that cysteine exhibits nanoenzyme inhibitory effect unlike other sulfur-containing amino acids. This makes the detection of cysteine with the as synthesized AgNPs selective. Even glutathione with the –SH group didn't show the inhibitory effect on the catalytic activity of the AgNPs. Structural differences between cysteine and glutathione ascribe to this observation.^55^ A relatively larger glutathione molecule with steric hindrance results in weak interaction between the sulfhydryl group and AgNPs.^53,56^ Whereas the strong interaction between the sulfhydryl group of cysteine and AgNPs inhibited the catalytic activity significantly that consequence a low absorption intensity of DAP. The results show high selectivity of the method in detection of cysteine.

### 3.6. Determination of cysteine in urine samples

The feasibility of the method for detecting cysteine in practical samples was investigated using human urine samples taken from a healthy individual. The urine sample was diluted with deionized water and spiked with specified concentrations of cysteine. The same procedure employed for detection of cysteine in deionized water was applied to the cysteine spiked urine sample. The catalytic activity of AgNPs using the non-spiked urine sample and water as a control were compared and a marginal inhibition in the catalytic activity of AgNPs was observed. The spiked urine samples of different concentrations of cysteine, however, showed comparative inhibiting property towards the peroxidase-like activity of AgNPs. The recovery ranges from 89.9 to 103.0%. The result shows the capacity of our method in detection of cysteine practically in real sample analysis (Table 2).

**Table 2:** Determination of cysteine in urine samples using our developed method

Urine	Spiked cysteine (μM)	Obtained (μM)	Recovery (%)
1	10	10.16	101.6
2	13	13.39	103.0
3	15	13.87	92.5
4	18	16.19	89.9

## 4. Conclusions

Uniformly dispersed AgNPs with peroxidase-mimic activity were synthesized successfully at room temperature using morula leaves extract as a reducing and capping agent. The method avoids utilization of toxic molecules and high temperature reaction. The obstruction of the catalytic activity of AgNPs in the presence of cysteine gives an opportunity to develop a colorimetric method for detection of cysteine. The strong interaction of AgNPs and cysteine makes the method specific to cysteine that will create opportunity for sensing of cysteine in different physiological fluids. Although biosynthesized plasmonic nanoparticles have been used for LSPR based colorimetric detection of various biomolecules, their applications as nanozymes have been inadequate. Therefore, this report paves a new avenue for utilization of biosynthesized plasmonic nanoparticles as nanozymes for colorimetric detection.

## Ethical statement

Informed consent was obtained from all human subjects.

## Author contributions

Melisew Tadele Alula: conceptualization, methodology, formal analysis, investigation, writing – original draft, review, and editing.

## Conflicts of interest

The author declares that he has no known competing financial interests or personal relationships that could have appeared to influence the work reported in this paper.

## Supplementary Material

RA-013-D3RA01587D-s001

## References

[cit1] Borase H. P., Patil C. D., Salunkhe R. B., Suryawanshi R. K., Kim B. S., Bapat V. A., Patil S. V. (2015). Appl. Biochem. Biotechnol..

[cit2] Li R. S., Zhang H. Z., Ling J., Huang C. Z., Wang J. (2016). Appl. Spectrosc. Rev..

[cit3] Tajik S., Dourandish Z., Jahani P. M., Sheikhshoaie I., Beitollahi H., Asl M. S., Jang H. W., Shokouhimeh M. (2021). RSC Adv..

[cit4] Niu Y., Ding T., Liu J., Zhang G., Tong L., Cheng X., Yang Y., Chen Z., Tang B. (2021). Talanta.

[cit5] Mokhtari A., Goudarzi A., Benam M., Langroodi S. M., Karimmohammad S., Keyvanfard M. (2016). RSC Adv..

[cit6] Zhang L., Lu B., Lu C., Lin J.-M. (2014). J. Sep. Sci..

[cit7] Chen S., Gao H., Shen W., Lu C., Yuan Q. (2014). Sens. Actuators, B.

[cit8] Wu L.-L., Wang L.-Y., Xie Z.-J., Pan N., Peng C.-F. (2016). Sens. Actuators, B.

[cit9] Saha A., Khalkho B. R., Deb M. K. (2021). RSC Adv..

[cit10] Han C., Xu K., Liu Q., Liu X., Li J. (2014). Sens. Actuators, B.

[cit11] Li Y., Li Z., Gao Y., Gong A., Zhang Y., Hosmane N. S., Shen Z., Wu A. (2014). Nanoscale.

[cit12] Ravindran A., Mani V., Chandrasekaran N., Mukherjee A. (2011). Talanta.

[cit13] Mazhani M., Alula M. T., Murape D. (2020). Anal. Chim. Acta.

[cit14] Liao H., Liu G., Liu Y., Li R., Fu W., Hu L. (2017). Chem. Commun..

[cit15] Chi Z., Wanga Q., Gu J. (2023). Analyst.

[cit16] Das B., Franco J. L., Logan N., Balasubramanian P., Il Kim M., Cao C. (2021). Nano-Micro Lett.

[cit17] Arshad F., Mohd-Naim N. F., Chandrawati R., Cozzolino D., Ahmed M. U. (2022). RSC Adv..

[cit18] Kavitha S., Mary Jelastin Kala S., Anand Babu Christus A., Ravikumar A. (2021). RSC Adv..

[cit19] Gao L., Zhuang J., Nie L., Zhang J., Zhang Y., Gu N., Wang T., Feng J., Yang D., Perrett S., Yan X. (2007). Nat. Nanotechnol..

[cit20] Feke K., Alula M. T. (2023). Spectrochim. Acta, Part A.

[cit21] Alula M. T., Madingwane M. L. (2020). Sens. Actuators, B.

[cit22] Wang J., Li W., Zheng Y.-Q. (2020). New J. Chem..

[cit23] Zhao X., Li S., Yu X., Gang R., Wang H. (2020). Nanoscale.

[cit24] Feke K., Alula M. T., Spende H., Waag A., Lemmens P. (2023). J. Cluster Sci..

[cit25] Alula M. T., Madingwane M. L., Yan H., Lemmens P., Zhe L., Etzkorn M. (2022). Environ. Sci. Pollut. Res..

[cit26] Jiang X., Xu W., Chen X., Liang Y. (2019). Anal. Methods.

[cit27] Wu G.-W., He S.-B., Peng H.-P., Deng H.-H., Liu A.-L., Lin X.-H., Xia X.-H., Chen W. (2014). Anal. Chem..

[cit28] Jiang H., Chen Z., Cao H., Huang Y. (2012). Analyst.

[cit29] Alula M. T., Feke K. (2023). J. Cluster Sci..

[cit30] Sun Z., Zhang N., Si Y., Li S., Wen J., Zhu X., Wang H. (2014). Chem. Commun..

[cit31] Roy A., Bulut O., Some S., Mandal A. K., Yilmaz M. D. (2019). RSC Adv..

[cit32] Bello B. A., Khan S. A., Khan J. A., Syed F. Q., Anwar Y., Khan S. B. (2017). J. Photochem. Photobiol., B.

[cit33] Khan M., Shaik M. R., Adil S. F., Khan S. T., Al-Warthan A., Siddiqui M. R. H., Tahir M. N., Tremel W. (2018). Dalton Trans..

[cit34] Rao B., Tang R.-C. (2017). Adv. Nat. Sci.: Nanosci. Nanotechnol..

[cit35] Cyril N., George J. B., Joseph L., Sylas V. P. (2019). J. Cluster Sci..

[cit36] Li S., Shen Y., Xie A., Yu X., Qiu L., Zhang L., Zhang Q. (2007). Green Chem..

[cit37] Nasrollahzadeh M., Issaabadi Z., Sajadi S. M. (2018). Sep. Purif. Technol..

[cit38] Ishak N. A. I. M., Kamarudin S. K., Timmiati S. N., Sauid S. M., Karim N. A., Basri S. (2023). J. Cleaner Prod..

[cit39] Fleischmann M., Hendra P. J., McQuillan A. J. (1974). Chem. Phys. Lett..

[cit40] Li C., Huang Y., Li X., Zhang Y., Chen Q., Ye Z., Alqarni Z., Bell S. E. J., Xu Y. (2021). J. Mater. Chem. C.

[cit41] Alula M. T., Mengesha Z. T., Mwenesongole E. (2018). Vib. Spectrosc..

[cit42] Huang Y.-F., Wu D.-Y., Zhu H.-P., Zhao L.-B., Liu G.-K., Ren B., Tian Z.-Q. (2012). Phys. Chem. Chem. Phys..

[cit43] Alula M. T., Lemmens P., Bo L., Wulferding D., Yang J., Spende H. (2019). Anal. Chim. Acta.

[cit44] Abdel-Lateef M. A. (2022). Sci. Rep..

[cit45] Fornera S., Walde P. (2010). Anal. Biochem..

[cit46] Vetr F., Moradi-Shoeili Z., Özkar S. (2018). Appl. Organomet. Chem..

[cit47] Wu L.-L., Wang L.-Y., Xie Z.-J., Pan N., Peng C.-F. (2016). Sens. Actuators, B.

[cit48] Sun Y., Wang J., Li W., Zhang J., Zhang Y., Fu Y. (2015). Biosens. Bioelectron..

[cit49] Farhadi K., Forough M., Pourhossein A., Molaei R. (2014). Sens. Actuators, B.

[cit50] Wu X.-Q., Xu Y., Chen Y.-L., Zhao H., Cui H.-J., Shen J.-S., Zhang H.-W. (2014). RSC Adv..

[cit51] Deng X., Zhao J., Ding Y., Tang H., Xi F. (2021). New J. Chem..

[cit52] Jiang C., Wei X., Bao S., Tua H., Wang W. (2019). RSC Adv..

[cit53] Huang J. T., Yang X. X., Zeng Q. L., Wang J. (2013). Analyst.

[cit54] Liyanage P. D., Weerathunge P., Singh M., Bansal V., Ramanathan R. (2021). Nanomaterials.

[cit55] Singh M., Weerathunge P., Liyanage P. D., Mayes E., Ramanathan R., Bansal V. (2017). Langmuir.

[cit56] Li W., Zhi X., Yang J., Zhang J., Fu Y. (2016). Anal. Methods.

