# Supplementary material for: Peroxidase-like activity of biosynthesized silver nanoparticles for colorimetric detection of cysteine
Source: RSC Adv. 2023 May 31;13(24):16396–404. doi: 10.1039/d3ra01587d (PMC10231313; doi:10.1039/d3ra01587d)
Supplement: RA-013-D3RA01587D-s001 [file RA-013-D3RA01587D-s001.pdf]

Electronic Supplementary Material (ESI) for RSC Advances.
This journal is © The Royal Society of Chemistry 2023

Peroxidase-like activity of biosynthesized silver nanoparticles for colorimetric detection of cysteine

Melisew Tadele Alula*

Department of Chemical and Forensic Sciences, Faculty of Science, Botswana International University of Science and Technology, Plot 10071, Private Bag 16, Palapye, Botswana

Supplementary data

Table S1. Qualitative phytochemical screening of leaves extract of *Sclerocarya birrea*

No	Secondary Metabolites	Type of test	Observation	Result
1	Steroids	Salkowski test	Brown precipitate upon standing	Positive
2	Terpenoids	Salkowski test	Reddish brown precipitate and golden yellow solution	Positive
3	Saponins	Foam test	Foam formation after 15 minutes	Positive
4	Alkaloids	Wagner's test	Yellow solution observed- no white precipitate	Negative
5	Sugars	Benedict's test	Orange-brick red precipitate was formed	Positive
6	Flavonoids	Ammonium test	Appearance of yellow color at ammonia layer	Positive
7	Tannins	Ferric chloride test	Black precipitate formed immediately	Positive
8	Phenols	Ferric chloride test	No green or blue color was observed	Negative

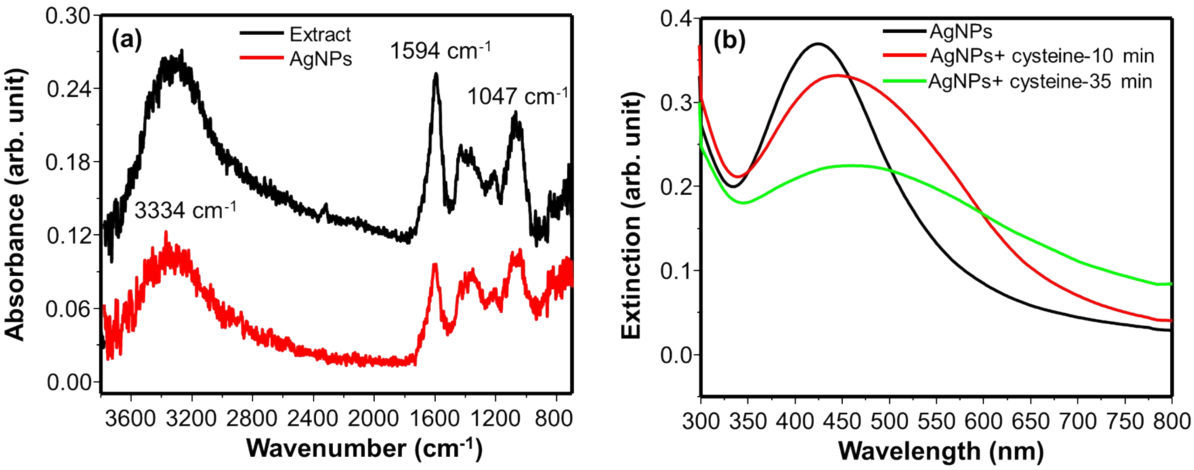

Fig. S1 (a) FTIR spectra of the *Sclerocarya birrea* (Morula) leaves aqueous extract and the synthesized AgNPs obtained using ATR mode. (b) Extinction spectrum of AgNPs colloids and AgNPs colloids mixed with 100 μM cysteine for 10 and 35 min.

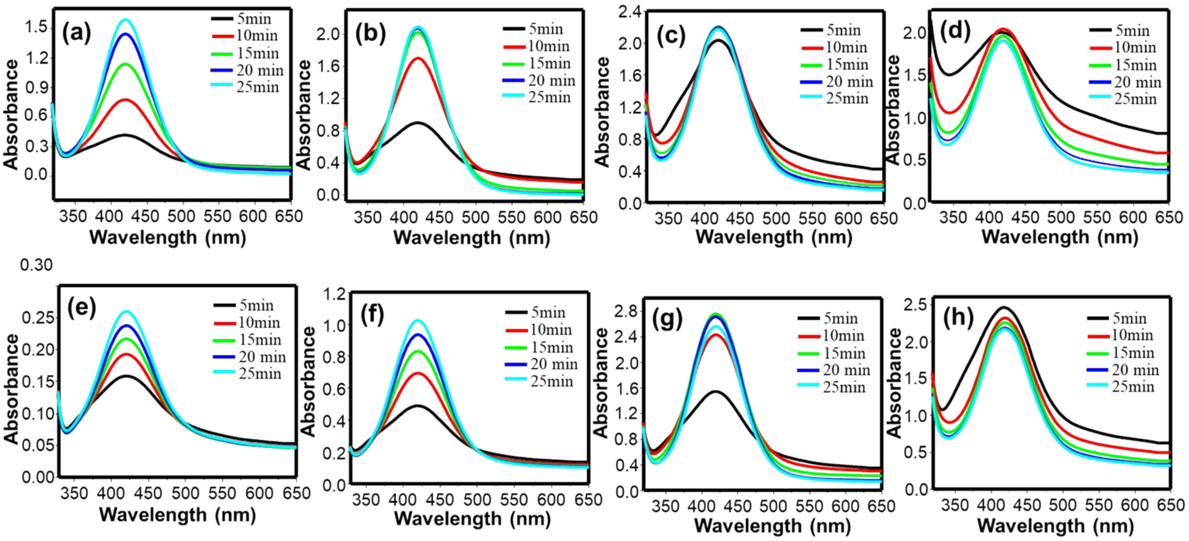

Fig. S2 Spectra of DAP in the absence of cysteine using different volume of AgNPs (a) 10 μL (b) 25 μL (c) 50 μL and (d) 75 μL and in the presence of cysteine using different volume of AgNPs (e) 10 μL (f) 25 μL (g) 50 μL (h) 75 μL.
